# Long-term snow alters the sensitivity of nonstructural carbohydrates of *Syntrichia caninervis* to snow cover: Based on a 7-year experiment

**DOI:** 10.3389/fpls.2022.999584

**Published:** 2022-10-14

**Authors:** Shujun Zhang, Qing Zhang, Ziyi Liu, Sulayman Mamtimin, Xiaobing Zhou, Benfeng Yin, Yuanming Zhang

**Affiliations:** ^1^ State Key Laboratory of Desert and Oasis Ecology, Xinjiang Institute of Ecology and Geography, Chinese Academy of Sciences, Urumqi, China; ^2^ Xinjiang Key Laboratory of Biological Resources and Genetic Engineering, College of Life Science and Technology, Xinjiang University, Urumqi, China; ^3^ Department of Geography, Economics and Geography-BSc(Econ), University College London, London, United Kingdom

**Keywords:** Biological soil crust, nonstructural carbohydrates, sensitivity index, climate change, soil physical and chemical properties

## Abstract

The dynamics of nonstructural carbohydrates (NSC) profoundly affect productivity and ecological adaptability to adversity in plants. Global warming induced the frequent occurrence of extreme precipitation events that altered the winter snow pattern in deserts. However, there is a lack of understanding of how desert mosses respond to long-term snow cover change at the NSC level. Therefore, in this study, long-term (7-years) winter snow removal (-S), ambient snow (CK), and double snow (+S) experiments were set in the field to investigate the content of NSC and its component in *Syntrichia Caninervis*. Our results showed that changes in snow depth, snow years, and their interaction significantly affected NSC and its component of *Syntrichia caninervis*. Compared to snow removal, NSC, soluble sugar, and starch significantly decreased with the increasing snow depth. The ratio of soluble sugar to starch significantly increased, while NSC and soluble sugar gradually returned to the normal level with an increase in snow years. It is worth mentioning that snow removal significantly reduced the soluble sugar to starch ratio compared to ambient snow depth, whereas the double snow experiment significantly increased the ratio of soluble sugar to starch during winter. This indicated an obvious trade-off between carbon utilization and carbon storage in *Syntrichia caninervis*. Snow removal stimulated *Syntrichia caninervis* to store sufficient carbon sources by starch accumulation for its future growth, while double snow promoted its current growth by soluble sugar accumulation. The variance in decomposition showed that soil physical and chemical properties, snow cover, and their interaction explained 83% of the variation in NSC and its components, with soil and plant water content, pH, and electrical conductivity (P-WC, S-WC, S-pH, and S-EC) as significant predictors. This highlights that snow indirectly affected NSC and its component contents by changing soil physical and chemical properties; however, long-term changes in snow cover could slow down its sensitivity to snow.

## Introduction

Biological soil crust is an organic complex formed by mutual cementation of soil particles, photoautotrophic (e.g., cyanobacteria, algae, lichens, mosses), and heterotrophic microorganisms (e.g., bacteria, fungi), which is widely distributed in arid and semi-arid regions, with the coverage of over 70% in some deserts (Belnap et al., 2008; Weber et al., 2022). Desert mosses, as an active component in the advanced stage of biological soil crust development, play an important role in enhancing surface stability, altering surface hydrological processes and vegetation succession (Zhao et al., 2014; Li et al., 2018; Rodriguez-Caballero et al., 2018). Unlike vascular plants, mosses have monolayer cells in their leaves, which are exceptionally sensitive to environmental changes and can restore photosynthesis in a short time after rehydration (Zhang et al., 2007; Zheng et al., 2011). Many studies have shown that changes in moisture conditions can alter moss growth and affect their physiological characteristics, nutrient cycling, and microbial activity (Belnap et al., 2004; Kidron et al., 2010; Chen and Duan, 2015). In the face of extreme environmental stresses such as drought, mosses can mitigate the damage by adjusting their physiological characteristics (Yin and Zhang, 2016; Li and Zhang, 2018; Li et al., 2021). Therefore, understanding the physiological mechanisms of mosses in response to water level changes is extremely important, particularly how climate change affects desert ecosystems.

NSC is one of the important physiological indicators to measure the response of plants to environmental changes (Song et al., 2022). NSC (including soluble sugar and starch) is the primary energy source during plant growth, metabolism, developmental, and reproduction processes. Its content reflects the dynamic balance between photosynthesis and respiration in plants (Hartmann and Trumbore, 2016; He et al., 2020; D'Andrea et al., 2021). Soluble sugar is primarily used to meet current energy demand and osmoregulation. Starch is the main storage form of photosynthetic products used to meet future energy demand. Moreover, soluble sugar and starch can transform into each other to withstand environmental stresses, so soluble sugar is negatively correlated with the starch content in the stress environment (D'Andrea et al., 2021). The change in NSC is the response of plants to environmental changes, which effectively alleviates the damage caused by environmental stress and regulates the growth and development of plants. Deslauriers et al. (2021) found that soluble sugar of the wild blueberry is transformed into starch to maintain normal physiological functions under winter conditions, and soluble sugar gradually decreased with the prolongation of snow cover during winter. In addition, an experiment on physiological effects of snow removal on sugar maple in New Hampshire, USA found that snow removal resulted in deepening of soil freezing, imbalanced soil cationic field, inhibited growth of terminal buds, and increased the content of NSC in leaves, which was mainly caused by increased starch content (Comerford et al., 2013). However, the response of the red spruce foliage NSC to the winter freeze-thawing cycle was not significant, indicating different plants had different responses to winter snow (Perkins and Adams, 1995). Nevertheless, there is a lack of research on how desert mosses would respond to changes in winter snow in terms of NSC, and the solution to this question would provide insight into the cold resistance properties of desert mosses.

Global warming has become an indisputable fact, which not only increases temperature but also significantly affects global precipitation patterns, especially the snow cover pattern during winter at mid-high latitudes (Group et al., 2007; Anderson and Gough, 2017; Wuebbles et al., 2017). Changes in winter snow cover could cause physiological stress on the plant, particularly during freezing-thawing periods (Bjerke et al., 2011; Yin et al., 2021). The existence of snow cover changes the soil microenvironment, slows down the number and intensity of freeze-thawing events, and avoids damage to the plants caused by water deficiency and low-temperature conditions (Tan et al., 2014; Fu et al., 2018; Hui et al., 2022). Given unabated global warming and the frequent occurrence of extreme climatic events, it is not clear how the changes in winter snow cover and the increase of snow years will affect the NSC of desert mosses. Therefore, we raise the following research question: what will be the *Syntrichia caninervis* NSC level response to snow cover changes over the years?

Based on the above problem, this study proposes the following hypotheses: (1) Low temperature and low moisture environment caused by snow removal will significantly improve the NSC content of *Syntrichia caninervis*, and it will increase significantly with snow cover years; (2) Snow removal will significantly increase the frequency of soil freeze-thawing cycles, breaking soil aggregates, and increasing soil rapid-acting nutrients, which will promote the accumulation of NSC. To verify the above scientific hypotheses, we selected the Gurbantungut Desert with stable snow cover during winter as a study area, and the dominant moss, *Syntrichia caninervis*, as the research object. In November 2014, 2017, and 2020, three snow gradients, including snow removal (-S), ambient snow (CK), and double snow (+S) were set up in the field to explore the NSC of *Syntrichia caninervis* after 7 years of continuous snow depth change. This study may provide an important reference for the stability of desert ecosystems under global climate change.

## Materials and methods

### Study site

The Gurbantunggut Desert (44°11′~ 46°20′ N, 84°31′~90°00′ E) is the largest fixed and semi-fixed desert dunes in China, located in the Jungger Basin of Xinjiang Uygur Autonomous Region, covering 4.88 × 10 4 km^2^. The altitude of the study region is 300–600 m amsl, the average annual precipitation is 70–150 mm, and the average annual evaporation is about 2000 mm, with a mean annual temperature of about 8°C (Zhang et al., 2007). This desert has a stable snow depth of about 15–20 cm during winter, and the snow period is maintained for about 100–150 days. The melting of snow during spring provides sufficient water for the germination and growth of ephemeral plants and annual herbs, which form a unique plant community in the region (Zhou et al., 2009). Additionally, well-developed biological soil crusts and vascular plants have become important landscapes on the desert surface (Li et al., 2017). As one of the typical dry mosses, *Syntrichia caninervis* is widely distributed in the area, which plays an important role in promoting soil stability and improving the surface microenvironment (Zhang et al., 2007).

### Experimental design

In 2014, we set the sample plots in a relatively homogeneous and flat inter-dune area in the center of the Gurbantunggut. The sample plots were enclosed with barbed wire to avoid human activities and animal interference. We set three snow gradients, snow removal (-S), ambient snow (CK), and double snow (+S) in a random distribution manner, and eight small (1.5 m×1.5 m) sample plots were set for each treatment. In addition, 24 snow treatment quadrat were set up in the same area in 2017 and 2020, with the same specifications as in 2014. Each quadrat was separated by buffer zones of at least 2 m wide to avoid variation due to different treatments. During snowfall, we placed 30 cm high steel barson in the snow removal treatment plots, and transparent acrylic sheets were placed above as a snow shielding material, while the surroundings were wrapped by ventilated nylon net to prevent wind-blown snow from drifting into the plots. After each snowfall event, the snow from the snow removal samples was evenly added to the double snow sample through a sieve. Finally, the nylon net and acrylic sheets were removed after the snow melted (Zhang and Zhang, 2020). After the snow, which completely melted in March 2021, five plots were randomly chosen to collect *Syntrichia caninervis* from the snow sample plots that were set up in 2014, 2017, and 2020. Stems and leaves of the aboveground part of *Syntrichia caninervis* were quickly removed using a sharp, frozen in liquid nitrogen and brought back to the laboratory for standby (Yin and Zhang, 2016).

### Water content

The water content of *Syntrichia caninervis* was measured by the drying method. Fresh moss samples were weighed (*W_1_*) accurately with an analytical balance. It was then oven-dried at 105°C for 15 min, and at 80°C for 48 h to get a constant weight (*W_2_*). The relative water content (*WC*) of *Syntrichia caninervis* was calculated as follows:


$$WC=\left(W_{1}-W2\right)/W1*100\%$$

### NSC analysis

In this study, NSC was considered as the sum of soluble sugars and starch contents which were measured by the anthrone-sulfuric acid method (Li et al., 2008).

Soluble sugars analysis: Dried samples were homogenized and pulverized to a fine powder using a ball mill. 0.1000 g was accurately weighed on an electronic scale (a precision of 0.0001 g). Then the powdered material was placed in a 10 mL centrifuge tube, and 5 mL of 80% ethanol was added. The mixture was incubated in a boiling water bath for 10 min, and then centrifuged at 4000 g for 10 min. The pellets were re-extracted twice with 80% ethanol and combined into the supernatant. After adding 5 mL anthrone solution to 1 mL of the extract, the mixture was shaken and heated in a 90°C water bath for 15 min, and allowed to cool. The absorbance was read at 620 nm, and the standard curve was used to determine the soluble sugar content.

Starch analysis: 10 mL of 30% *perchloric acid was added to the remaining pellets* overnight. The mixture was incubated at 80°C for 10 min and then centrifuged at 4000 g for10 min. The supernatant for the samples was transferred into a 50 mL volumetric flask and kept at a constant volume with distilled water. After adding 5 mL anthrone solution to 1 mL of the extract, the mixture was shaken, heated in a boiling water bath for 10 min, and allowed to cool. The absorbance was read at 620 nm, and the standard curve was used to determine the starch content.

### Data analysis

To visualize the response of NSC and its to different snow depths and snow years, the following data were analyzed by subtracting the control group from the treatment group. Data of the NSC of the *Syntrichia caninervis* were tested before analyses for normality and homogeneity. Differences in snow depth, snow years, and their interaction were assessed using *repeated measures ANOVA*. Differences in NSC and its components between treatments were evaluated using a *repeated measures analysis of *variance and multiple comparisons (LSD). All the tests were performed using SPSS 20.0 statistical packages for Windows (SPSS Inc., Chicago, IL, USA) and mappings were done with Origin 20.0 (Origin Lab, Northampton, Massachusetts, USA).

The influences of NSC, soluble sugar, starch, and the ratio of soluble sugars to starch were analyzed by using Spearman’s correlation analysis and random forest model. Relations between the prime abiotic factors and NSC, soluble sugar, starch, and their ratio were analyzed by regression analysis to explore potential abiotic factors that affected NSC and its components. Besides, variance in decomposition of snow, soil physical and chemical properties, and plant nutrients were used to explore the contribution of NSC and its components. All tests were performed using R 4.1.3 software, mainly using the packages of *random Forest*, *link ET*, and *tidyverse*.

To visualize the response of NSC and its components to different snow depths and snow years, we introduced sensitivity analysis to evaluate the sensitivity index *(SI)* of NSC to different snow depths and snow years. The specific equation is as follows:


SI=Vt−Vn

*Vt* is the measured value of different snow depths; *Vn* is the measured value of ambient snow.

## Results

### Water content

**Figure 1:**
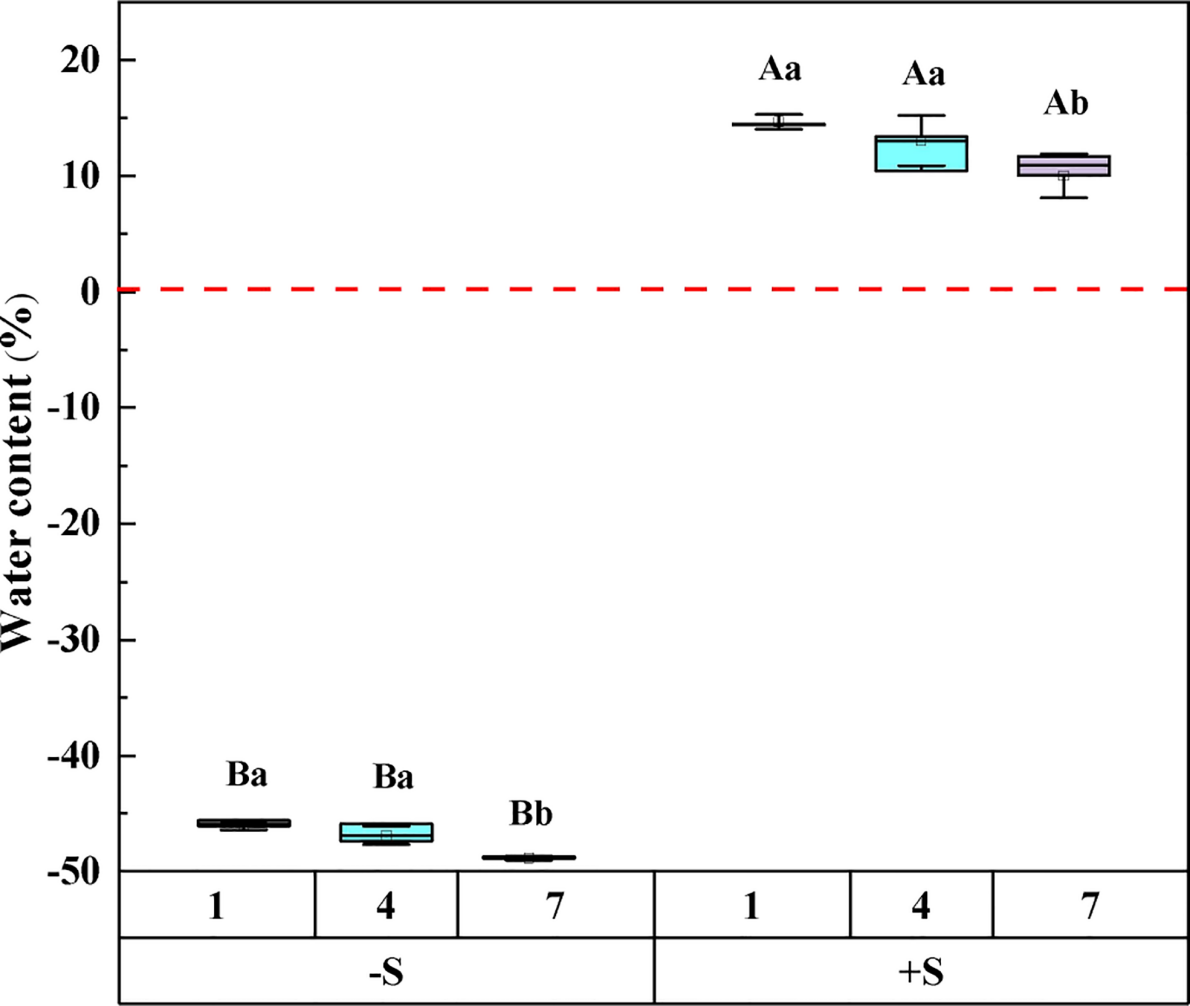
The effect of snow depth and snow years on the water content of *Syntrichia caninervis*. -S represents snow removal, +S represents double snow. Data are means ± SEM, n = 5. Different capital letters and lowercase letters indicate significant differences (P < 0.05) between treatments of snow depth and snow years, respectively.

The results of *repeated* *measures ANOVA* showed that snow depth, snow years, and their interaction significantly affected the water content of *Syntrichia caninervis* (**Table 1**). Compared to snow removal, the plant water content increased significantly with an increase in snow depth. With the increase in the snow years, the water content of the plants under the two snow treatments decreased gradually (**Figure 1**).

**Table 1:** Repeated* *measures* *ANOVA of snow depth and snow years on the content of NSC and its components of *Syntrichia caninervis*.

Factor	P-WC	SS	ST	SS/ST	NSC
	F-values	F-values	F-values	F-values	F-values
Snow	6317.725**	82.618**	788.609**	131.179*	1381.697**
Years	7.914*	8.277**	7.392*	5.184*	0.365
Snow ×Years	0.961	0.557	17.088**	1.618	10.08**

** indicate p < 0.01; * indicate p < 0.05. Plant water content (P-WC), soluble sugar (SS), starch (ST), the ratio of soluble sugars to starch (SS/ST), nonstructural carbohydrates (NSC).

### NSC and its components of *Syntrichia caninervis*

**Figure 2:**
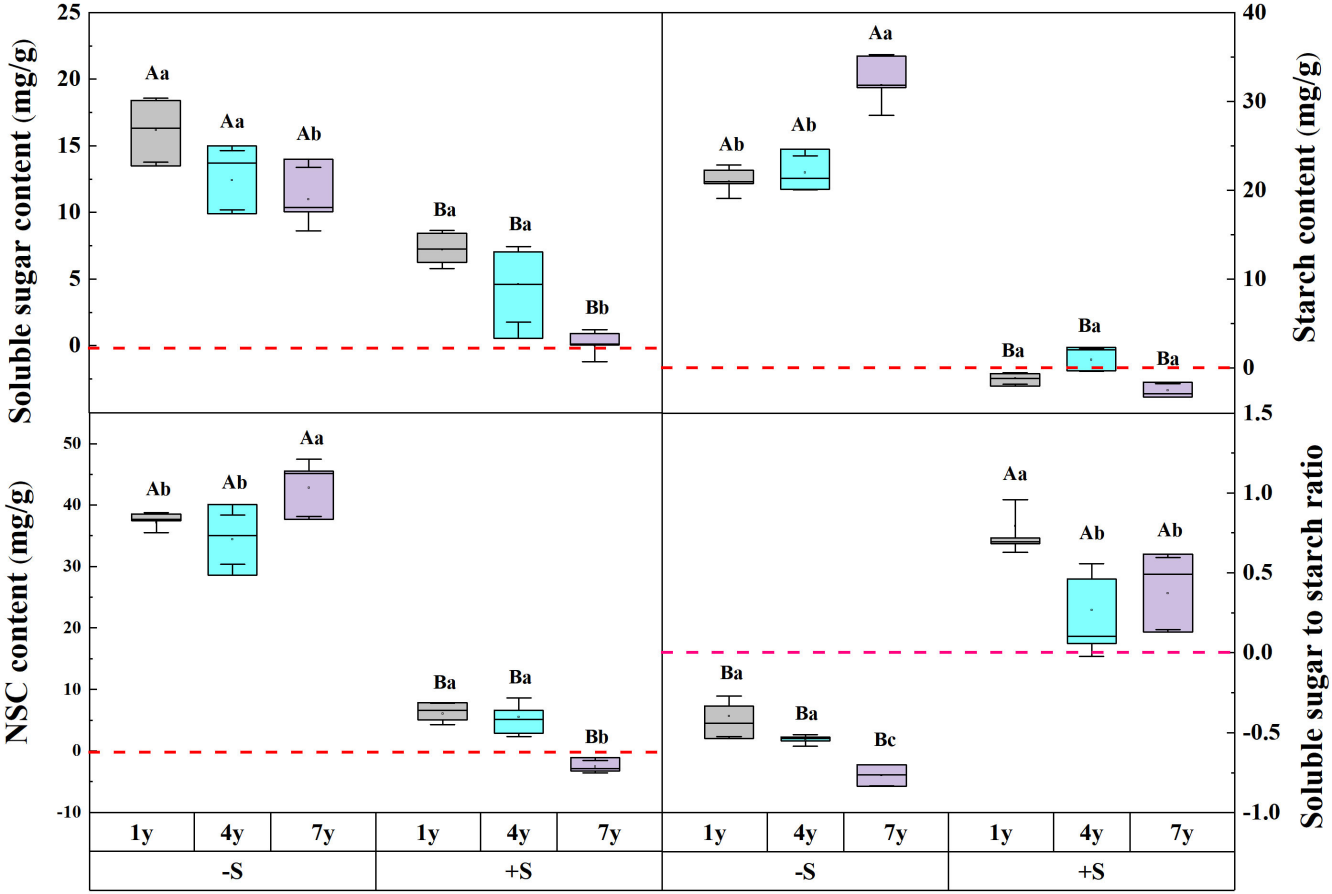
The effect of snow depth and snow years on the NSC and its components of *Syntrichia caninervis*. Data are means ± SEM, n = 5. Different capital letters and lowercase letters indicate significant differences (P< 0.05) between treatments of snow depth and snow years, respectively.

The snow depth, snow years, and their interaction significantly affected the contents of NSC and its components (**Table 1**). Snow removal significantly increased NSC, soluble sugar, and starch (*p*<0.05). However, the degree of increase was affected by the years of snow removal, indicating a decline in soluble sugar, while the contents of NSC and starch showed an upward trend. Compared to ambient snow, an increase in snow significantly increased NSC and soluble sugar. With the increase of snow years, soluble sugar decreased gradually and attained ambient snow level. Snow removal decreased the ratio of soluble sugar to starch, and it decreased year by year with the increase in snow removal years. Compared to ambient snow, the double snow significantly improved the soluble sugar to starch ratio, and gradually attained a natural level with an increase in snow years (**Figure 2**).

### Potential driving factors of NSC and its components content of *Syntrichia caninervis*

**Figure 3:**
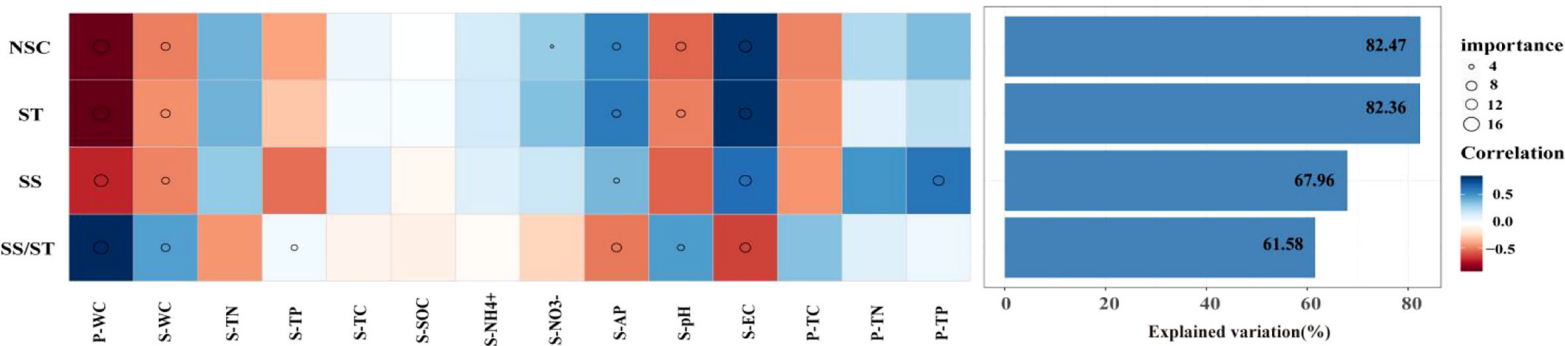
Contributions of abiotic factors to the NSC and its components of *Syntrichia caninervis* based on Spearman’s correlation and random forest model. Circle size represents the variables’ importance (i.e., percentage of increase of mean square error calculated *via* random forest model). Colors represent Spearman’s correlations. Plant water content (P-WC), soil water content (S-WC), soil electrical total nitrogen (S-TN), soil total phosphorus (S-TP), soil total carbon (STC), soil organic carbon (S-SOC), ammonia nitrogen ( $S-NH_{4}^{+}$ ), soil nitrate nitrogen ( $S-NO_{3}^{-}$ ), soil available phosphorus (S-AP), soil Ph (S-pH), soil conductivity(EC), plant total carbon(P-TC), plant electrical total nitrogen (P-TN), plant total phosphorus (P-TP).

**Figure 4:**
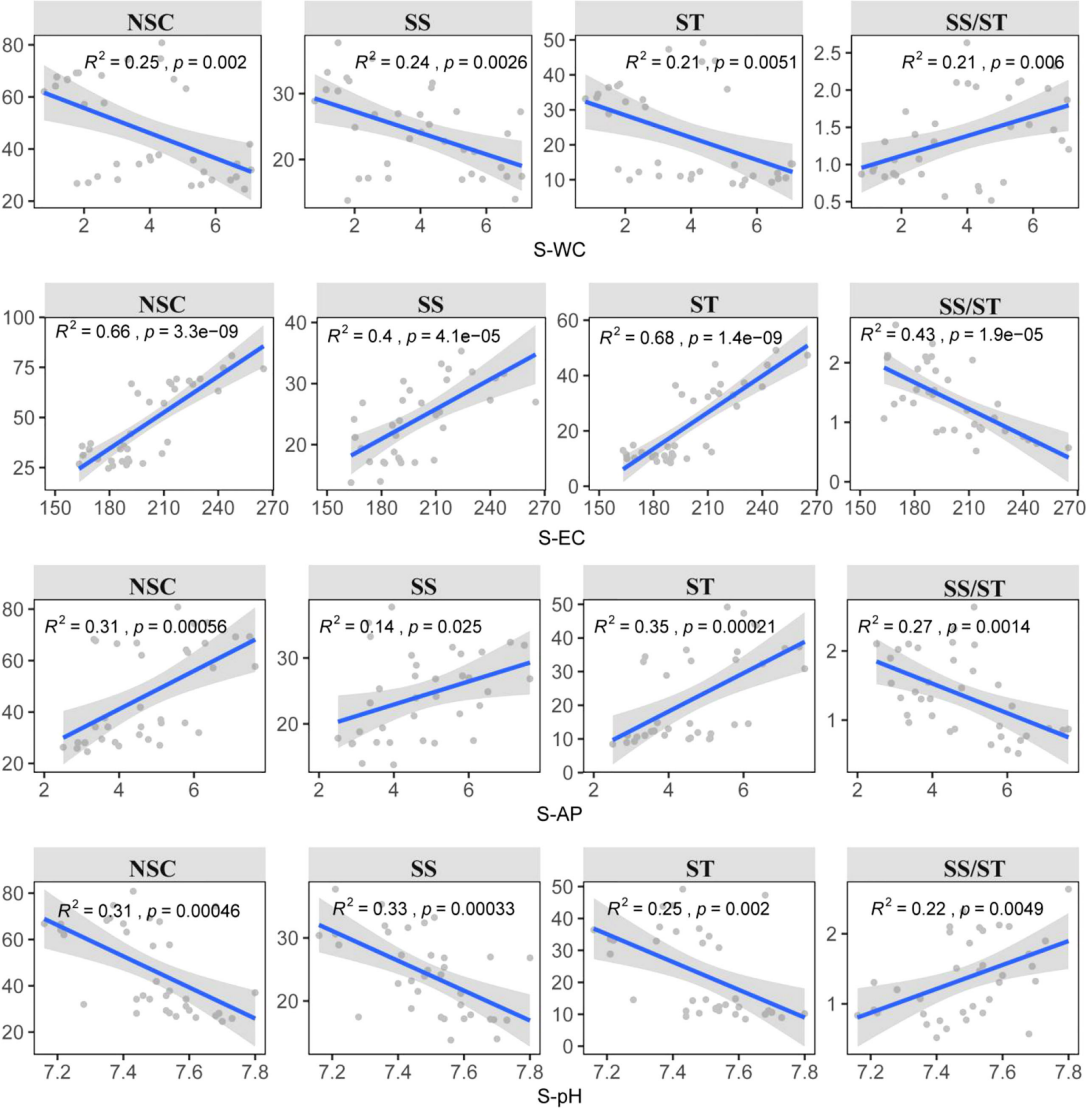
Relationships between the NSC and its components of *Syntrichia caninervis* and the main drivers were estimated *via* linear least‐squares.

The results showed that there were differences in the key factors affecting NSC and its component contents. The abiotic factors explained more variation in NSC and starch (82.47%, 82.36%) than in soluble sugar and the ratio of soluble sugar to starch (67.96%, 61.58%). P-WC, S-WC, S-AP, S-pH, and S-EC were important predictors explaining the variations in NSC and its component contents (**Figure 3**). Among them, P-WC and S-EC contributed the most to explaining the variations (**Figure 3**). This result was further confirmed by a significant negative correlation between P-WC and NSC, while the content of NSC and its components increased with increase in S-EC. In contrast, the ratio of soluble sugar to starch showed the opposite trend (*p*< 0.05) (**Figure 4**). Secondly, P-TP was also one of the key factors affecting soluble sugar content, and there was a significant negative correlation between P-TP and soluble sugar content (*p *< 0.05) (**Figure 4**). P-WC and S-WC had a significant positive correlation with the ratio of soluble sugar to starch, while S-AP and S-EC were negatively correlated with it (*p*< 0.05) (**Figure 4**).

**Figure 5:**
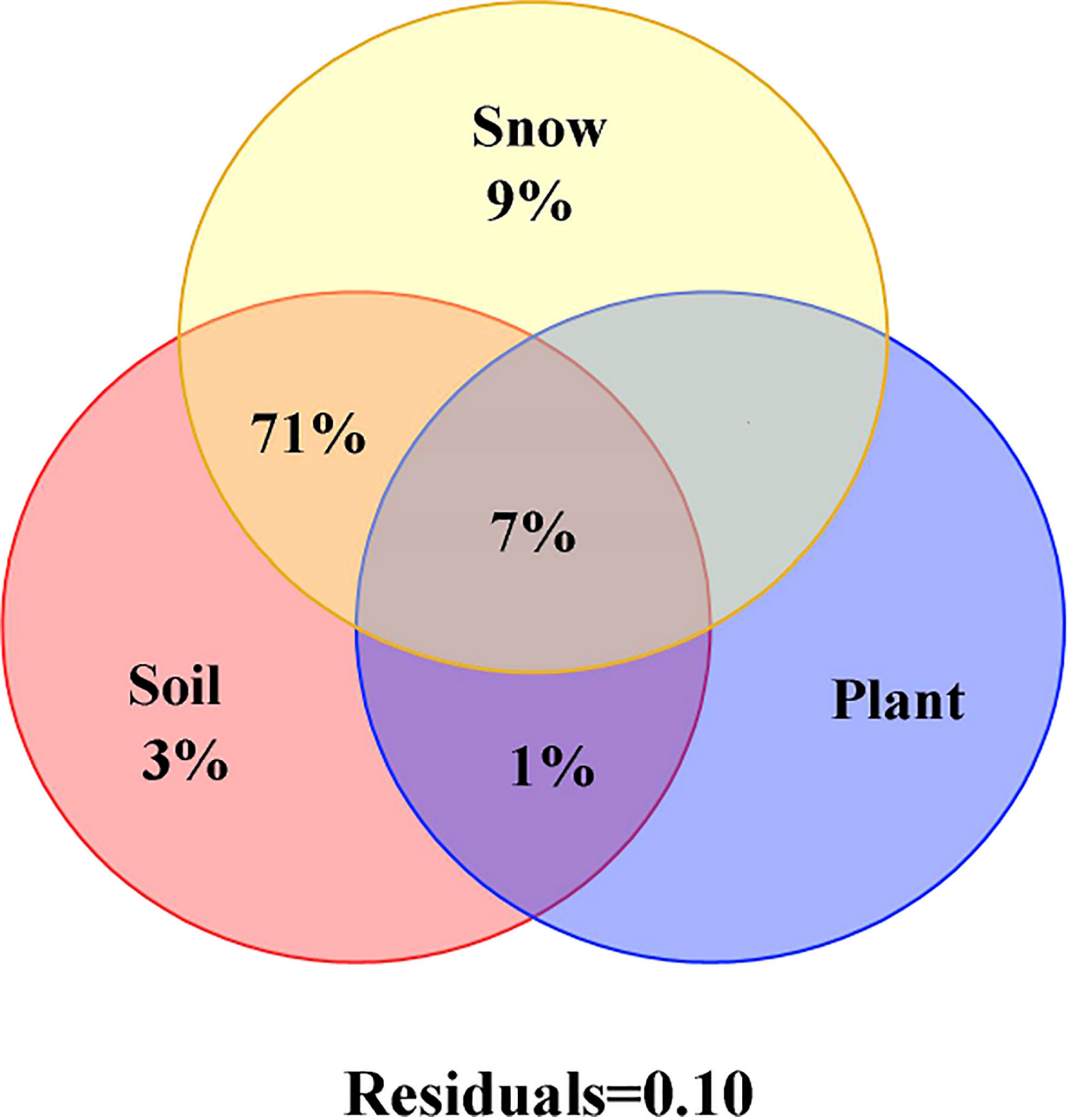
Influence factors of NSC, soluble sugar, starch and the ratio of soluble sugar to starch of *Syntrichia caninervis* on 7-year continuous snow cover change by the variance decomposition analysis.

The variance in decomposition showed that NSC and its component contents changed with 7-years of continuous snow depth changes. It accounted for 71% and was mainly explained by the interaction between snow and soil physical and chemical properties (**Figure 5**). The individual effect of snow accounted for 9% of the variation in NSC and its component contents, whereas the individual effect of soil physical and chemical properties explained just 3%. The explanation rates of individual effects of the plant nutrients were among the lowest (**Figure 5**).

**Figure 6:**
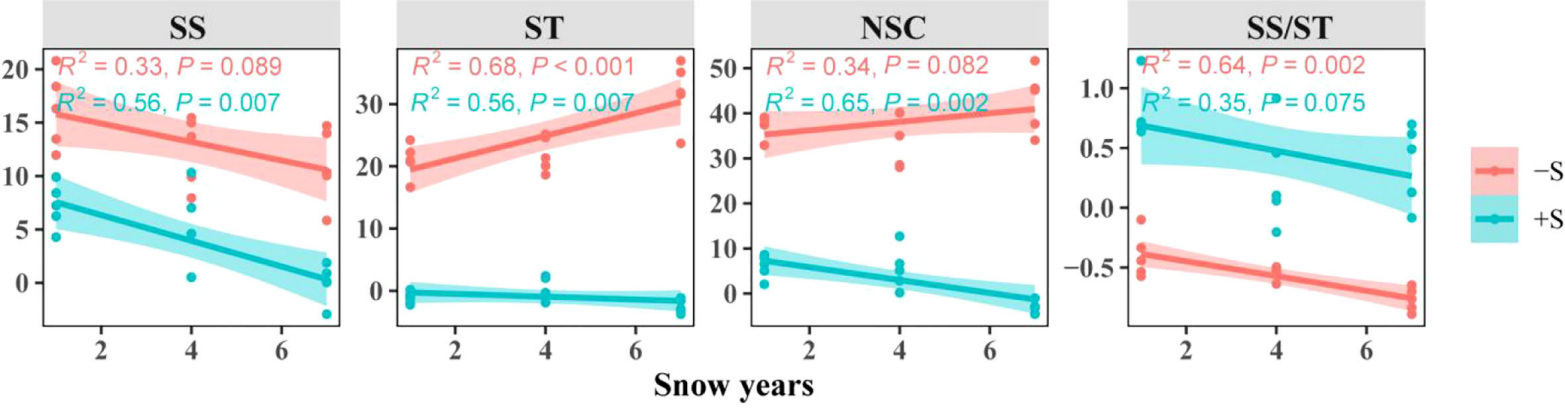
Sensitivity characteristics of *Syntrichia caninervis* to snow cover under different snow years.

It can be seen from **Figure 6** that the sensitivity of NSC and its components of *Syntrichia caninervis* to snow elevated with increasing snow years. The shorter the number of snow years, the more sensitive the response of *Syntrichia caninervis* to snow. However, the sensitivity of different indexes to snow and their responses to snow years varied. The soluble sugar and the ratio of soluble sugar to starch were the most sensitive to snow and snow years, while the starch was the least sensitive.

## Discussion

### Effect of snow on the NSC and its component contents of *Syntrichia caninervis*

Snow removal significantly increased NSC of *Syntrichia caninervis*, and the NSC increased year by year with years of snow removal, which is consistent with our first hypothesis. Our results indicated that *Syntrichia caninervis* stored the energy fixed by photosynthesis to cope with the environmental stresses caused by drought and low temperature under the condition of snow removal. This observation verifies the “carbon sink limitation hypothesis” of plants in a low-temperature environment (Korner, 2003; Shi et al., 2019). This could be explained as follows: the removal of snow caused drought, low temperature, and a high radiation environment, which tended to decouple growth and photosynthesis in *Syntrichia caninervis*. This inhibited growth, which resulted in the restriction of carbon sink so that it prolongs the survival rate of plants during stress through the accumulation of NSC (Muller et al., 2011; O'Brien et al., 2014; O'Brien et al., 2015). NSC accumulation not only provided a carbon source for the growth of *Syntrichia caninervis*, but also played a role of buffer in the absence of photosynthesis (Hoch et al., 2003). Compared with ambient snow, the double snow experiment provided an environment with sufficient water. The increase of NSC content reduced the osmotic potential and helped to maintain the normal turgor, and contributed to the normal progress of photosynthesis and cell growth. The increase of NSC was mainly due to the increase of soluble sugar (Quick et al., 1992; Qian et al., 2018; Gersony et al., 2020). However, with the extension of the double snow years, the NSC content decreased year by year and attained the natural level. This result indicated that the effect of environmental stresses on NSC had a temporal pattern (He et al., 2020). During the long period of snow doubling, NSC was regarded as a metabolic substance for the growth of *Syntrichia caninervis*, and concurrently acted as an osmotic regulatory substance during photosynthesis. This resulted in its content decreasing yearly and gradually returning to a natural level (Li and Zhang, 2018).

In this study, we found that the *allocation model of NSC of Syntrichia caninervis* changed by the snow cover and snow years. The soluble sugar is the prime energy source and osmotic adjustment substance, and the plant could enhance its resistance ability by increasing the soluble sugar under environmental stress such as low temperature and drought (Klopotek and Klaering, 2014; Rodriguez-Calcerrada et al., 2011; Rodriguez-Caballero et al., 2018). Winter snow forms a thermal insulation layer to alleviate the damage caused by the low temperatures, while the melting of snow provides sufficient water for the mosses to decrease the soluble sugar in the spring. Thus, it slows down the water loss, maintains the stability of the membrane proteins, and prevents oxidative damage under low temperatures in the one-year snow experiment (Dietze et al., 2014; Yin and Zhang, 2016; Hui et al., 2018). We confirmed this observation in this study, where soluble sugar of *Syntrichia caninervis* significantly increased with a decrease in snow. This is mainly because the degree of suffering from environmental stresses, such as low temperature, low water, and high radiation, deepened with a decrease in snow, which caused it could enhance cell concentration and water-holding capacity of *Syntrichia caninervis*. Thus, by the accumulation of soluble sugar, *Syntrichia caninervis* avoided growth arrest caused by cell damage (Yin et al., 2021). However, Compared with one year’s snow treatment, the long-term changes in the snow led to a cold adaptation mechanism in *Syntrichia caninervis*, which reduced its sensitivity to environmental stresses, such as low temperature and moisture, and therefore soluble sugar content was decreased year by year (Zhang and Zhang, 2020; Deslauriers et al., 2021).

In this study, the hostile environment created due to snow removal caused a sharp reduction in soluble sugar content that could promote the accumulation of starch content to maintain normal physiological function (O'Brien et al., 2015). Starch content showed an increasing trend with snow removal years for specific performance. This may be because the soluble sugar supply was sufficient to maintain normal growth and osmotic regulation under these conditions, which resulted in excessive soluble sugar conversion into starch, and reserved a large amount of carbon source for the recovery of *Syntrichia caninervis* (Adams et al., 2013). Concurrently, a change in the soluble sugar to starch ratio also verifies this assumption. Under the condition of snow removal, the ratio of soluble sugar to starch was significantly lower than the ambient snow level decreased year by year with an increase in snow removal years. It shows that the increase in starch is much greater than the decrease in soluble sugar under this condition. The starch of *Syntrichia caninervis* did not change significantly with snow years under double snow treatment. This underlined that the *Syntrichia caninervis* tended to synthesize soluble sugar to meet its own needs under the condition of double snow cover, but it tended to store the energy fixed by the photosynthesis to cope with the environmental stress under the condition of extreme snow removal. This distribution pattern reflects that the *Syntrichia caninervis* chose a conservative and low-cost survival strategy in the face of 7-year of continuous snow removal.

### Effect of soil on NSC and its component contents of *Syntrichia caninervis*

Snow depth and snow years significantly changed the content of NSC and its components by changing the physical and chemical properties of the soil. Due to low temperature and low water environment caused by the removal of snow, available nutrient content of the soil and NSC of *Syntrichia caninervis* increased significantly, which is consistent with our second hypothesis. First, snow removal increased the frequency of soil freezing-thawing cycle, and large aggregates were cleaved into small aggregates in the soil (Sulkava and Huhta, 2003; Wang et al., 2022). At the same time, low temperature led to the cracking and death of a large number of microorganisms, which increased S-EC and available nitrogen. This promoted the absorption of N by *Syntrichia caninervis*, increased chlorophyll content and net photosynthetic rate, and helped in accumulating NSC (Baxter et al., 1992; Tomassen et al., 2003; Kim et al., 2012; Zhang et al., 2020). Compared with snow removal, the persistence of snow slowed down the time and intensity of soil freezing and thawing, resulting in small changes in daily temperature, which promoted physiological activities, consuming part of NSC, and reducing NSC content (Steinweg et al., 2008; Zhao and Jin, 2020). Compared to ambient snow, double snow treatment increased microbial activity, which enhanced the mineralization of C, N, and P in the soil, resulting in lower organic nutrient content and higher inorganic nutrient content and promoted NSC accumulation (Xu et al., 2011). This indicates that snow depth and snow years caused changes in soil available nutrient content by affecting the N and P mineralization process, which led to changes in the NSC.

Snow removal decreased S-pH and increased S-EC and soil available nutrients, increased soluble sugar and starch content, and decreased the ratio of soluble sugar to starch. This result was that snow removal caused deep soil freezing, increasing $NO_{3}^{-}$ , which led to an increase in soluble sugar (Xing et al., 2020). In addition, the snow removal promoted soil acidification, reduced soil pH, and soil enzyme activity. It affected the absorption and utilization of soil nutrients by *Syntrichia caninervis*, which reduced photosynthesis, and inhibited the growth of *Syntrichia caninervis*. It caused the accumulation of soluble and starch (Boutin and Robitaille, 1995; Tomlinson, 2003; O'Brien et al., 2015). This result also validated the “growth limitation hypothesis” of plants in low-temperature environments. The degree of soil acidification was deepened, and nutrient content in the plants was reduced with an increase in snow removal years. This resulted in excessive soluble sugar converted into starch for storage, providing a sufficient carbon source for the recovery of *Syntrichia caninervis* in the future (Knox and Clarke, 2005). Our result also verifies that the ratio of soluble sugar to starch was lower than the natural level under snow removal treatment and showed a decreasing trend year by year. In addition, an increase in double snow significantly affected soluble sugar content compared to ambient snow, but the starch level was not significantly changed. This is mainly due to an increase in soil nutrient content and microbial activity caused by snow, which drove microbial decomposition of the *Surfactant* of *Syntrichia caninervis* and provided the ability to fix and store nutrients *Syntrichia caninervis*, increasing the. Increased N content in the soil and plant* protein *synthesizing* capacity* promoted enzyme activities related to photosynthesis and carbon metabolism, which in turn increased soluble sugar content, facilitated water use and transport, and enhanced carbon utilization strategy (Schimel et al., 2004; Garnett et al., 2009; Mo et al., 2020; Wang et al., 2022). However, long-term double snow treatment led to a decrease in soluble sugar content and gradually attained natural levels. This might be because an increase in soluble sugar content inhibited excessive $S-NO_{3}^{-}$ under long-term snow conditions. It could also be possible that recovery of S-pH and S-EC promoted a decline in soluble sugar to natural levels.

## Conclusion

The continuous warming of the global climate and the frequent occurrence of extreme climate caused significant changes in snow patterns in desert winter. The 7-years continuous snow depth change significantly affected the content of NSC and its components of *Syntrichia caninervis*. Compared to the snow removal, the content of NSC and its components of *Syntrichia caninervis* significantly decreased with the increase of snow depth. However, the sensitivity of NSC of *Syntrichia caninervis* to snow depth and that to snow years was different. The soluble sugar and the ratio of soluble sugar to starch were the most sensitive, while the starch was insensitive. In conclusion, the Syntrichia caninervis accumulated starch in the snow removal environment, while soluble sugar accumulated in the double snow environment, and the physiological sensitivity of *Syntrichia caninervis* to snow depth was changed with the increase of snow years.

## Data availability statement

The original contributions presented in the study are included in the article/supplementary material. Further inquiries can be directed to the corresponding authors.

## Author contributions

BY, XZ and YZ planned and designed the research. SZ and BY analysed data and wrote the manuscript. QZ and ZL performed experiments. All authors contributed to the article and approved the submitted version.

## Funding

This research was supported by the National Natural Science Foundation of China (41901134, U2003214), the Youth Innovation Promotion Association of the Chinese Academy of Sciences (2020437), the China Postdoctoral Science Foundation (2019M653805).

## Conflict of interest

The authors declare that the research was conducted in the absence of any commercial or financial relationships that could be construed as a potential conflict of interest.

## Publisher’s note

All claims expressed in this article are solely those of the authors and do not necessarily represent those of their affiliated organizations, or those of the publisher, the editors and the reviewers. Any product that may be evaluated in this article, or claim that may be made by its manufacturer, is not guaranteed or endorsed by the publisher.
